# Environmentally Realistic Exposure to the Herbicide Atrazine Alters Some Sexually Selected Traits in Male Guppies

**DOI:** 10.1371/journal.pone.0030611

**Published:** 2012-02-01

**Authors:** Kausalya Shenoy

**Affiliations:** Department of Biology, University of Kentucky, Lexington, Kentucky, United States of America; University of Akron, United States of America

## Abstract

Male mating signals, including ornaments and courtship displays, and other sexually selected traits, like male-male aggression, are largely controlled by sex hormones. Environmental pollutants, notably endocrine disrupting compounds, can interfere with the proper functioning of hormones, thereby impacting the expression of hormonally regulated traits. Atrazine, one of the most widely used herbicides, can alter sex hormone levels in exposed animals. I tested the effects of environmentally relevant atrazine exposures on mating signals and behaviors in male guppies, a sexually dimorphic freshwater fish. Prolonged atrazine exposure reduced the expression of two honest signals: the area of orange spots (ornaments) and the number of courtship displays performed. Atrazine exposure also reduced aggression towards competing males in the context of mate competition. In the wild, exposure levels vary among individuals because of differential distribution of the pollutants across habitats; hence, differently impacted males often compete for the same mates. Disrupted mating signals can reduce reproductive success as females avoid mating with perceptibly suboptimal males. Less aggressive males are at a competitive disadvantage and lose access to females. This study highlights the effects of atrazine on ecologically relevant mating signals and behaviors in exposed wildlife. Altered reproductive traits have important implications for population dynamics, evolutionary patterns, and conservation of wildlife species.

## Introduction

The role of sex hormones in the expression of sexually selected traits has been established in many vertebrate species, especially in males [1], [2], [3], [4], [5]. Disruption of the expression or perception of such traits can influence mate choice and evolutionary patterns [6], [7], [8], [9]. The increase in various forms of pollution is becoming an important factor in such disruptions [6], [10] and is hence instrumental in shaping evolutionary trajectories. A common form of pollution is caused by endocrine disrupting compounds (EDCs), which interfere with proper hormonal functioning. These compounds can be natural or synthetic in origin, including organochlorines, organophosphates, polychlorinated biphenyls (PCBs), phthalates, synthetic hormones and hormone-blockers, and phytoestrogens. Many of them have anthropogenic sources such as pesticides, industrial effluents, pulp mill effluents, plastics and sewage. Significant routes of exposure include direct exposures from living in contaminated soil or water, as well as indirect exposures through eating contaminated prey [11], [12], [13], [14]. EDCs can alter reproductive success by affecting all aspects of the reproductive system, including gonadal formation, production of hormones and gametes, sex determination [15], formation of egg shells [16], and production [15], [17] and maintenance of mating signals and behaviors [18], [19].

The effects of EDCs on wildlife have been receiving increasing attention in the literature in recent years. While earlier toxicological studies focused on mortality effects from acute exposures, ecotoxicologists are now focusing on sub-lethal effects of more realistic exposures. Sub-lethal effects can be subtle yet far-reaching by influencing population and community dynamics through cascading effects. Population level effects may include altered demographics [20], [21], [22], [23] and mating systems [24], [25], [26], [27]. This can affect community dynamics by impacting species closely associated with the focal species. Multi-generational effects due to persistence of pollutants in the environment across generations, or via maternal transfer, can affect evolutionary trajectories of these species as a result of altered sex ratios and mating systems.

The current study focused on the effects of atrazine, a widely used triazine herbicide. Atrazine is the second most commonly used pesticide in the US [28]. It is resistant to degradation, and its half-life in surface waters can be over 700 days [29], [30]. Many animal species that spend all or part of their life cycle in water can be exposed to significant levels of the chemical for a considerable part of their life. Concentrations of atrazine in water bodies around agricultural fields are expected to be in the range of 19–194 ppb (90 day average) depending on the type of crop and application rate [31]. Non-target species inhabiting water bodies around agricultural fields are particularly at risk for exposure to atrazine. Atrazine induces aromatization of testosterone to estradiol [32], [33], thereby causing an estrogenic effect in exposed individuals; however, this mechanism has been debated [34]. Several studies have demonstrated the feminizing effects of atrazine in amphibians [35], [36], [37], [38], yet the number of studies with ambiguous and conflicting results [39], [40] contributes to preventing policy changes regarding the use of this pesticide.

Here, I tested whether prolonged exposure to atrazine can alter male mating signal expression, including ornamentation and mating behaviors. I used guppies (*Poecilia reticulata*) as a model organism to test these questions, as guppies have distinct sexual dimorphism, their mating signals and behaviors have been well characterized [41], and the role of sex hormones in the expression of these traits has been explored [5], [42]. Further, guppies have been used for testing similar questions in other ecotoxicological studies [43], [44], [45]. Guppies are small tropical fish native to Trinidad and parts of South America. They are especially useful for testing hypotheses related to sexual selection. Males have different colored spots on their body and fins [41]; they perform characteristic courtship displays (called “sigmoid” displays) and attempt forced copulations. Mating is predominantly through female mate choice; females respond to courtship displays and to males with larger and brighter orange spots [41], [46], but avoid forced copulatory attempts [41], [47].

Although the pattern and intensity of orange spots are mostly governed by genetics [48], [49], there is some indication that androgens are required for their expression [43], [45], [48], [50], [51], [52], as well as for performing courtship displays [43], [50], [53]. Shenoy and Crowley [9] discuss in detail how hormones may be involved in the expression of sexual signals. An aromatase inducer like atrazine can alter hormonal balances by (1) increasing the estradiol concentrations, which would increase the estradiol: testosterone ratio, and directly reduce the production of testosterone [54], [55], and by (2) reducing the concentration of testosterone available for conversion to 11-keto testosterone [56], [57], an important teleost androgen required for the expression of secondary sexual characteristics.

I hypothesized that prolonged exposure to environmentally relevant doses of atrazine would (1) reduce the area and intensity of orange color spots, which are the primary male mating signals in guppies; (2) reduce the frequency of mating behaviors such as courtship displays and forced copulatory attempts (these were considered behaviors related to mating effort); and (3) in the presence of competing males, reduce the frequency of behaviors related to mating effort and those related to male-male aggression. The third hypothesis was tested because male-male competition is high in many animal species, including guppies, and examining behaviors in the context of mate competition is ecologically relevant. Further, contaminants are often differentially distributed in the landscape, and different individuals in a population may be exposed unequally; often, species that are migratory or that converge at breeding sites would have differentially exposed individuals within a population. Since individuals impacted to varying degrees would be competing together within a population, I tested the third hypothesis by pairing treated males with those that were not exposed to the contaminants. This also standardized the condition of each experimental male's opponent.

## Methods

### Ethics statement

The experimental protocol for this study was approved by the University of Kentucky Institutional Animal Care and Use Committee (protocol number 2007-0137).

### Treatments

85 guppies were randomly assigned to one of five treatments at 17 fish per treatment. The treatments included a control (no treatment), dimethylsulfoxide (DMSO, 6 ppb) as the solvent control, atrazine low-dose (1 ppb), atrazine high-dose (15 ppb), and ethynyl estradiol (2 ppb) as the estrogenic positive control. A solvent control was used because atrazine and ethynyl estradiol were dissolved in DMSO; all treatments received the same concentration of DMSO. Atrazine concentrations used were based on US EPA estimated environmental concentrations [31]. Pilot experiments helped determine sub-lethal ethynyl estradiol concentrations. Concentration of atrazine in the water column in three randomly selected jars per treatment was ascertained by liquid phase extraction with methylene chloride following an adaptation of US EPA Method 619 [58]—which produced 95% recovery of the target compound—and analyzed by gas chromatography/mass spectrometry. The average concentration at the end of one week was determined to be 0.26 ppb and 12.98 ppb for the low- and high-dose respectively, with negligible loss over the 7 days. No atrazine was detected in the control samples. Atrazine (98% purity) was purchased from Chem Service, Inc., through Fisher Scientific, and 17 α-ethynylestradiol (98% purity) was purchased from Sigma-Aldrich. Treatments continued for 16 weeks to simulate a long-term exposure.

### Animals

Adult male guppies used for this study were descendants of wild-caught guppies from Trinidad. Three populations—Aripo Upper River, Aripo Lower River, Guanapo Upper River—were equally represented in all treatments to account for geographic and genetic variation. All males included showed clear color patterns and gonopodium development [41], indicating sexual maturity. During the period of the study, all fish were housed separately in individual glass jars with 1.6 L of aged, pre-aerated, carbon filtered, conditioned water. Tropical fish flake food was fed once each day in *ad libitum* quantities. Room temperature was maintained at an average of 25°C; the light: dark cycle was set to 12∶12 hours. Water was changed once weekly with static renewal of chemical treatments. Mortality was recorded every day.

### Color measurements

All fish were photographed once before the start of treatments and once after treatments stopped with a Nikon D50 digital SLR camera with a 55 mm telephoto lens and Nikon SB-400 AF Speedlight flash. The shutter speed was set to 1/60 s, aperture to 22 F and film speed to 200 ISO. The flash speed was set to 1/16 s and power to −0.7, and was covered with a single sheet of tissue paper to diffuse the light. All fish were photographed on the left side in the same position relative to the lens and flash. ImageJ 1.43u [59] was used to measure the area of orange spots and body area of each fish in mm^2^. An average value of the red (R), green (G) and blue (B) channels of each orange spot was also measured. Each fish was photographed along with an orange color standard, which was placed in the same position in every picture. Colors were standardized across all pictures by applying a correction factor to each of the average R, G, B values, such that the corrected R value of the fish in the picture to be measured, R_i_′ = R_i_ * R_Sr_/R_Si_, where R_i_ is the average R value of the fish in the picture to be measured, R_Sr_ is the average R value of the color standard on one picture chosen to be the reference picture; R_Si_ is the average R value of the color standard on the picture to be measured. Similarly, G_i_′ and B_i_′ were calculated for each picture. A dark orange spot would have a high R′ measure, and lower G′ and B′ measures; on the other hand, a pale orange spot would have high R′, G′ and B′ measures. The repeatabilities of the corrected R′, G′ and B′ values were r = 0.98, r = 0.95 and r = 0.96, respectively. Further, a single composite variable comprising of all three color channels was created by inputting the corrected R′, G′, B′ values in a Principal Components Analysis and extracting one variable. The repeatability of this composite variable was found to be r = 0.98.

### Behavior trials

At the end of the 16 week treatment period, the fish were subjected to two sets of behavior trials: the first set assessed behavior of the males towards a female in the absence of competition from another male, and the second set of trials assessed mating behaviors in the presence of a competing male. All trials were conducted within the first four hours after lights turned on and during the last four hours before lights turned off. All trials were conducted blind: the observer did not know the treatment that any of the fish had received and identified males by their color patterns only. Data were recorded in real time. The observer sat in darkness, 1 m away from the tank, to avoid startling the fish; the fish did not appear to notice or be disturbed by the presence of the observer. Trial tanks were illuminated with full spectrum light to ensure that all colors were perceived naturally by the other fish in the trial [60], [61].

#### Trials without competing males

Each male was placed in a trial tank of dimensions 30×20×15 cm (height×length×width) and 7.5 L of water, with one virgin female from the same population. Water used was aged, pre-aerated, carbon filtered, and conditioned, and water temperature was maintained between 23–25°C. After a 5 minute acclimation period, the fish were observed for 10 minutes. The total number of sigmoid courtship displays, gonopodium swings and mating attempts were recorded throughout the trial period. Males frequently swing their gonopodium forward, and this appears to increase in frequency during mating or aggressive interactions; any gonopodium swing greater than 90° was counted.

#### Trials with competing males

These trials were conducted to test whether treatments altered male behaviors compared to an untreated male in the context of competition. Males were paired in the following fashion—each pair consisted of one male from the control group (opponent) and one male (focal male) from one of the other four treatment groups: DMSO, atrazine low-dose, atrazine high-dose, or ethynyl estradiol. Control group males were used in multiple pairs as there were not enough males to be used only once. Control group males were paired with each of the different treatment group males in random order. Males of a pair belonged to the same population. Pairs could not be size matched after matching for population; treatment group males were on average 14% of body area (8.32 mm^2^) larger or smaller than paired control group males. Body size was measured as area rather than length because this was a more realistic measure of what competing males would perceive. Each pair was placed in a trial tank of dimensions 30×20×15 cm (height×length×width) and 7.5 L of water, with a virgin female from the same population. After a 5 minute acclimation period, behaviors were recorded for 10 minutes. At each 10 s point, I recorded which male was closer to the female. A male had to be more than one body length ahead of the other male to be “closer”, and received 1 point in such cases. If both males were within one body length of each other, and within at least two body lengths of the female's vent, they were both recorded as being equally close; in such cases both males received 0.5 points. If both males were further than two body lengths from the female's vent, they were both recorded as being far from the female and received 0 points for that event. At the end of the 10 minute trial period, each male's “closeness” points were summed and its ratio to the total number of events gave a measure of proximity. Throughout the whole trial period, I counted for each male the total number of sigmoid courtship displays, mating attempts, aggressive displays to the rival male, and attacks on the other male. The number of gonopodium swings was not recorded, as these happened in quick succession, and the observer could not keep a reliable count for both males.

### Data Analyses

All data were analyzed in SAS 9.2 [62]. All statistical procedures refer to SAS procedures.

#### Mortality

Univariate survival analyses (lifetest procedure) were first used to test which variables (among treatment and population of origin) were to be included in the final model to test for effects on mortality. Based on the log-rank test of equality over strata, population of origin was not included in the model (χ^2^ = 3.096, p = 0.38). Difference in mortality between treatments was then analyzed using regression analysis of survival data based on the Cox proportional hazards model (phreg procedure).

#### Area of Orange Spots, Intensity of Orange Spots, and Mating Behaviors in the Absence of Competition

The dependent variables were appropriately transformed to meet the assumptions of parametric tests wherever required. Pearson's product-moment correlations between the measures of color and mating behaviors were analyzed using the corr procedure. A mixed model anova (mixed procedure) was used to analyze the treatment effects on (1) *Area of orange spots*: the change in proportion of orange between initial and final readings, (2) *Intensity of orange spots*: the change between initial and final readings of corrected R′, G′, and B′ values, and the composite variable, and (3) *Mating* **behaviors in the absence of competition.** The number of courtship displays and number of mating attempts. The correlation coefficients revealed that the number of gonopodium swings was correlated strongly with the number of courtship displays (*r* = 0.62, *P*<0.0001) and weakly with the number of mating attempts (*r* = 0.26, *P* = 0.04), and so this variable was eliminated from further analyses. A mixed model anova using the mixed procedure allows the use of fixed and random factors in the model; the effect of random factors, wherever included in the model, is removed and results are based on least square means that are adjusted for this effect.

Preliminary analyses determined that the control group and solvent control group did not significantly differ from each other for all variables and so the two groups were pooled as a common control group (area of orange spots, *P* = 0.9; R′, *P* = 0.22; G′, *P* = 0.22; B′, *P* = 0.81; composite variable, *P* = 0.27; number of courtship displays, *P* = 0.9; number of mating attempts, *P* = 0.08). Population of origin was input as the random effect wherever it improved the fit of the model as determined by significantly lower Akaike Information Criteria values (henceforth AIC statistics). For behavioral responses in the absence of competition, the identity of the female used for the trial (because females were used in multiple trials) was also included as a random factor, and time of day that the trial was conducted was included as a covariate, wherever these improved the fit of the model as determined by AIC statistics. Planned orthogonal contrasts were used to test whether (1) the atrazine low-dose and high-dose had similar effects on the response variables, (2) the two atrazine groups had significantly different effects on the response variables compared to the pooled control group, and (3) ethynyl estradiol had the strongest effect on the response variables compared to the other groups. One-tailed p-values were reported for these tests because of the clear directionality of the hypotheses. Further, Tukey's post-hoc tests were used to see which groups differed significantly from each other. Effect sizes with 95% confidence intervals of the differences between each of the treatment groups and the pooled control group were calculated as per Nakagawa and Cuthill [63].

#### Mating behaviors in the presence of competition: The dependent variables were appropriately transformed to meet the assumptions of parametric tests wherever required

Pearson's product-moment correlations between all variables were analyzed using the corr procedure. Due to the moderate correlations between some of the response variables (see Table 1 for correlations), and because all behaviors recorded on a pair of fish occurred during the same trial period, a manova was conducted with the glm procedure using the focal male's responses from each pair. Covariates and random effects were not included as the glm procedure is not equipped to handle these additional effects. Each response variable was then analyzed separately. Since males were paired, and their behaviors were dependent on each other, an ancova was performed with the mixed procedure to analyze the effect of the treatments on the focal male's behavior in response to his paired opponent's behavior, which was included as the covariate. Covariates were mean-centered within treatments so that mean estimates for each treatment corresponded with the mean value of the covariate. I specifically tested for differences between treatment intercepts (seen by a significant effect of the treatment) and slopes (seen by a significant interaction of treatment by covariate). A negative effect of the treatments on competitiveness would be indicated by a reduced slope and intercept of the relationship described above, compared to the DMSO (solvent control) group. The difference between the competing males in body size (measured by area of body in mm^2^) and proportion of body area covered by orange were input as additional covariates if they improved the fit of the model as determined by AIC statistics. Similarly, population of origin and control male's identity (because males from the control group were used in multiple pairs) were input as random effects wherever they improved the fit of the model. Further, each treatment-control paired data set was analyzed separately for each treatment (DMSO, atrazine low-dose, atrazine high-dose or ethynyl estradiol) with a paired design to test whether the treatment male consistently behaved differently from his paired control opponent, depending on what the treatment was. A mixed model anova (mixed procedure) was used to test this, with the pair identity input as a random effect with compound symmetry as the covariance structure. Population of origin was also input as a random effect wherever it improved the fit of the model as determined by AIC statistics. Time of day that the trial was conducted, the control male's trial number, the differences in body size and proportion of body area covered by orange between the competing males were input as covariates if they significantly improved the fit of the model.

**Table 1:** Pearson's correlation coefficients between variables of mating behavior in the presence of competing males.

	Proximity	Courtship displays	Mating attempts	Attacks
Courtship displays	***r*** ** = 0.512** (*P* = 0.0002)			
Mating attempts	***r*** ** = 0.396** (*P* = 0.0059)	***r*** ** = 0.398** (*P* = 0.0056)		
Attacks	*r* = −0.129 (*P* = 0.3874)	*r* = −0.081 (*P* = 0.5887)	*r* = 0.134 (*P* = 0.3676)	
Aggressive displays	***r*** ** = −0.368** (*P* = 0.0110)	*r* = 0.170 (*P* = 0.2522)	*r* = 0.0341 (*P* = 0.8196)	***r*** ** = 0.411** (*P* = 0.0041)

## Results

### Mortality

There were no significant effects of the treatments on mortality rate (likelihood ratio test: *χ* ^2^ = 6.87, df = 4, *P* = 0.14). The ethynyl estradiol group had the highest mortality over the 16 week period (47.06%) but the hazard ratio was not significantly higher than the control group (Hazard ratio = 3.38, *P* = 0.07). The mortality in the other groups was as follows: control 17.65%, DMSO 29.41%, atrazine low-dose 23.53%, and atrazine high-dose 11.76%. At the end of the exposure period, the number of surviving fish in each of the groups was: control = 14, DMSO = 12, atrazine low dose = 13, atrazine high dose = 15, ethynyl estradiol = 9.

### Color

**Figure 1:**
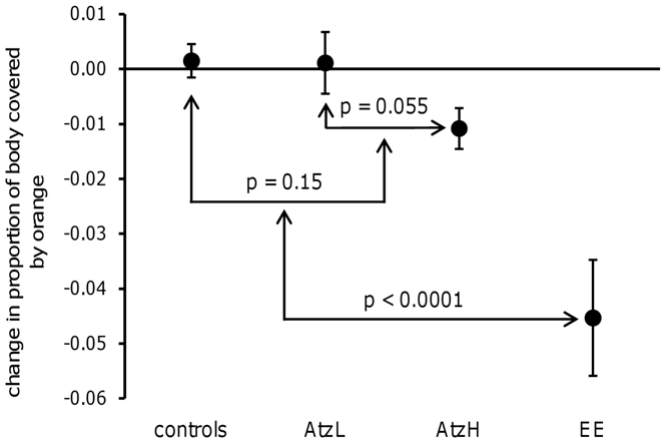
Treatment effects on change in proportion of body area covered by orange. Negative numbers suggest reduction in area of orange, while positive numbers suggest increase in area of orange. Treatments are labeled as follows: pooled control+DMSO group = “controls”, atrazine low-dose = “AtzL”, atrazine high-dose = “AtzH”, ethynyl estradiol = “EE”. Arrows between groups denote planned orthogonal contrasts.

The treatments had a significant effect on the change in body area covered by orange (*F*_3, 58_ = 14.19, *P*<0.0001; figure 1). This effect was mainly driven by the ethynyl estradiol group, which had a significantly lower proportional area of orange than the pooled controls (*P*<0.0001, effect size±95% confidence interval [d±95% CI] = −2.27±0.89), and all the other groups combined (planned orthogonal contrasts, p<0.0001). The atrazine high-dose appeared to reduce the area of orange (d±95% CI = −0.76±0.68, figure 1), but this was not statistically significant (P = 0.098).The atrazine low-dose did not reduce the area of orange (d±95% CI = −0.12±0.65), and the two atrazine groups differed from each other (planned orthogonal contrasts, *P* = 0.055, figure 1). Because of the difference between the two atrazine groups, they did not collectively reduce the area of orange compared to the pooled control group (planned orthogonal contrasts, *P* = 0.15). The Tukey's post-hoc tests brought out significant differences only between the ethynyl estradiol group and each of the other groups. The loss of power resulting from all pair-wise comparisons lead to a lack of statistical evidence for a difference between the atrazine high-dose and pooled control groups (unadjusted *P* = 0.04, Tukey's adjusted *P* = 0.14). The treatments did not affect the change in corrected R′ (*F*_3, 57_ = 0.29, *P* = 0.83), G′ (*F*_3, 57_ = 0.53, *P* = 0.67), and B′ (*F*_3, 57_ = 0.19, *P* = 0.90) values, or the composite variable (*F*_3, 57_ = 0.31, *P* = 0.82). The planned orthogonal contrasts did not reveal any significant patterns. Population of origin failed to improve the fit of the model for explaining the variation in body area covered by orange or corrected R′, G′, B′ and the composite variable, suggesting that this factor was not important in explaining the change in color over the study period.

### Mating behaviors in the absence of competing males

**Figure 2:**
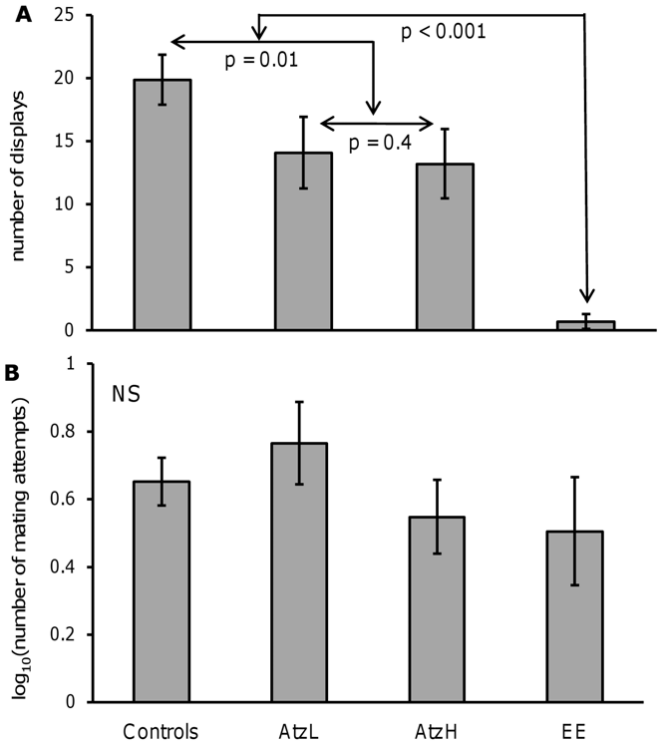
Treatment effects on mating behaviors. (A) The number of courtship displays performed to a female per 10 minute trial, and (B) the number of mating attempts per 10 minute trial. Treatments are labeled as follows: pooled control+DMSO group = “controls”, atrazine low-dose = “AtzL”, atrazine high-dose = “AtzH”, ethynyl estradiol = “EE”. Arrows between groups in panel A denote planned orthogonal contrasts. These are not shown for panel B because none of the contrasts were significantly different.

The number of mating attempts was weakly but negatively related to the proportion of body area covered by orange (*r* = −0.25, *P* = 0.046); there were no other significant correlations between any of the other measures of color and behavioral variables. The number of courtship displays differed significantly between treatments (*F*_3, 61_ = 9.79, *P*<0.0001; figure 2). The planned orthogonal contrasts determined that the ethynyl estradiol group displayed significantly less than the other groups (*P*<0.0001). The two atrazine groups displayed similarly to each other (*P* = 0.40), and together they displayed significantly less than the pooled controls (*P* = 0.01). The effect sizes showed that the ethynyl estradiol group displayed less than the pooled control group (d±95% CI = −2.15±0.65), as did the atrazine high-dose group (d±95% CI = −0.64±0.64), but the atrazine low-dose group did not display less than the pooled control group (d±95% CI = −0.56±0.65). The Tukey's post-hoc tests revealed similar trends, though the lack of power weakened some of these results. The number of mating attempts did not differ between groups (*F*_3, 58.1_ = 2.01, *P* = 0.12), and none of the planned orthogonal contrasts showed significant differences. Population of origin improved the fit of the model to explain variation in courtship display rates, but not the number of mating attempts.

### Mating behaviors in the presence of competing males

The measures of mating effort were all moderately correlated with each other (proximity to the female and number of courtship displays: *r* = 0.51, *P* = 0.0002, number of courtship displays and number of mating attempts: *r* = 0.40, *P* = 0.0056, proximity to the female and number of mating attempts: *r* = 0.40, *P* = 0.0059; Table 1), and the measures of aggression were also moderately associated with each other (the number of aggressive displays and the number of attacks on paired male: *r* = 0.41, *P* = 0.004; Table 1). Further, the proximity to a female was negatively associated with the number of aggressive displays (*r* = −0.37, *P* = 0.01; Table 1), and this is because males do not focus on the female during aggressive interactions and can often be far from her. The treatments had a significant effect on the focal males' responses as a whole (Wilks' λ = 0.42, *F*_15, 119.11_ = 2.95, *P* = 0.0005).

**Figure 3:**
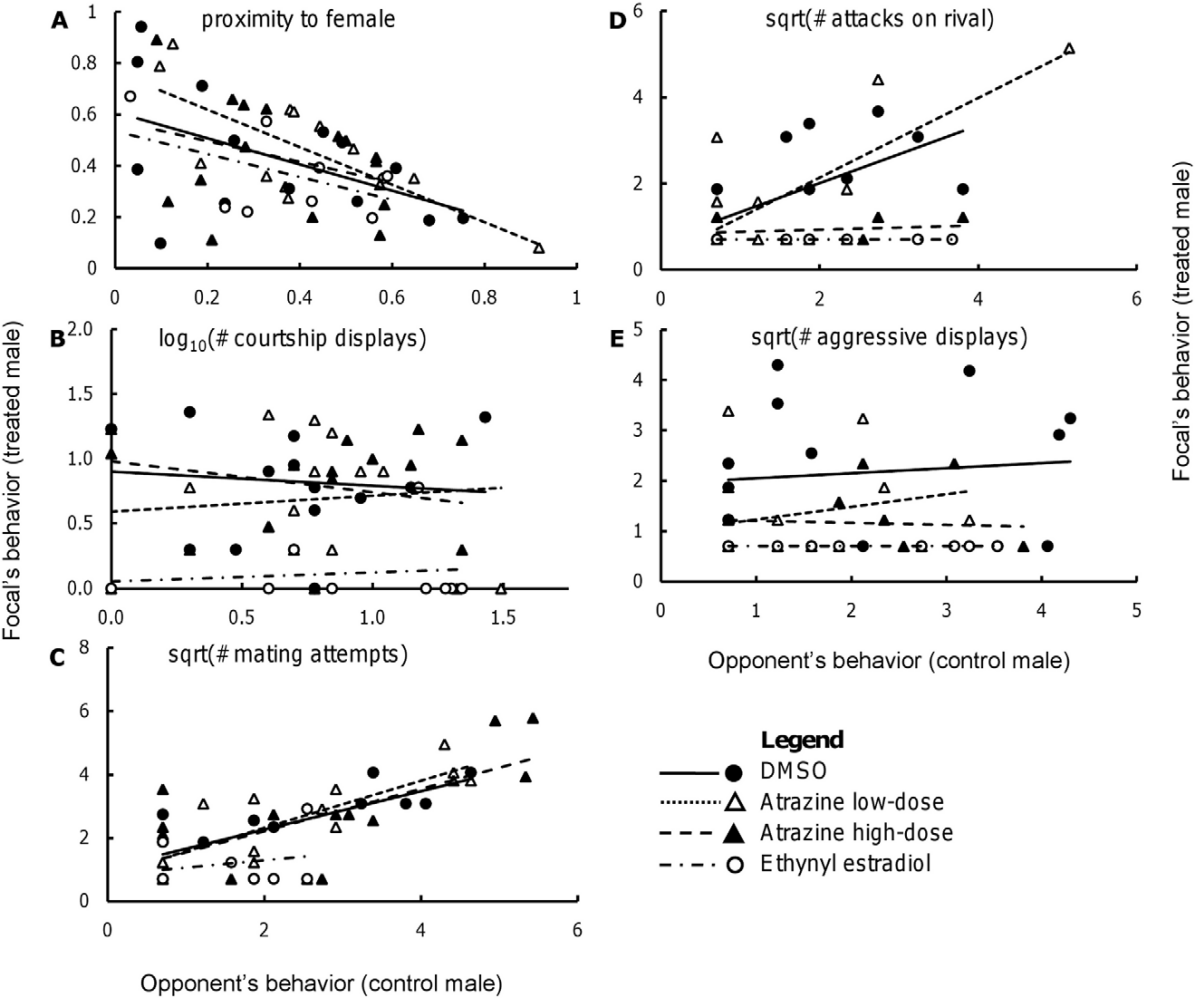
Treated males' behaviors in relation to paired control males' behaviors. Treated males—those belonging to DMSO, atrazine low-dose, atrazine high-dose and ethynyl estradiol groups—were the focals, while the paired male from the control group was the opponent. For each response variable, the x- and y-axes have the same measure and units. Results of the ancova corresponding to each panel: (A) Proximity: treatment, *F*_3, 36.7_ = 2.71, *P* = 0.059; opponent's response, *F*_1, 37.1_ = 5.47, *P* = 0.025; treatment×opponent's response, *F*_3, 36.9_ = 0.65, *P* = 0.59; (B) number of courtship displays: treatment, *F*_3, 39_ = 4.71, *P* = 0.007; opponent's response, *F*_1, 39_ = 0.01, *P* = 0.92; treatment×opponent's response, *F*_3, 39_ = 0.23, *P* = 0.88; (C) number of mating attempts: treatment, *F*_3, 39_ = 5.63, *P* = 0.0026; opponent's response, *F*_1, 39_ = 19.97, *P*<0.0001; treatment×opponent's response, *F*_3, 39_ = 0.18, *P* = 0.91; (D) number of attacks: treatment, *F*_3, 28.9_ = 8.25, *P* = 0.0004; opponent's response, *F*_1, 33.1_ = 14.41, *P* = 0.0006; treatment×opponent's response, *F*_3, 28.4_ = 10.37, *P*<0.0001; (E) number of aggressive displays: treatment, *F*_3, 30_ = 4.10, *P* = 0.015; opponent's response, *F*_1, 34.9_ = 0.72, *P* = 0.40; treatment×opponent's response, *F*_3, 32.4_ = 0.06, *P* = 0.98.

The treatments did not have significant effects on the proximity or number of mating attempts, or their interaction with their opponent's behaviors; the treatments significantly influenced the number of displays, but this was driven by the effect of ethynyl estradiol rather than either of the atrazine groups (figure 3a, b, c; table 2). Population of origin improved the fit of the model explaining variation in proximity and number of courtship displays, but not the number of mating attempts. The number of mating attempts was influenced by the difference in body size between the competing males (*P* = 0.04); the larger the focal male was compared to his paired control opponent, the more mating attempts he made. There was a significant effect of the treatment (*F*_3, 28.9_ = 8.25, *P* = 0.0004) and the interaction of treatment and covariate (*F*_3, 28.4_ = 10.37, *P*<0.0001) on the number of attacks on the competing male (figure 3d). The atrazine high-dose and ethynyl estradiol treatments significantly reduced the slopes and intercepts of the regression lines between the focal male's behavior and the paired control male's behavior (table 2) compared to the DMSO group. Treatments also affected the number of aggressive displays made to the rival male (*F*_3, 30_ = 4.1, *P* = 0.015; figure 3e) but had no effect on the interaction of treatment and covariate as there was no significant effect of the covariate itself. Both these variables were also influenced by the identity of the paired control male.

**Table 2:** Intercept and slope estimates of the treated males' behaviors in relation to those of the paired control males for each treatment group, as generated by the ancova.

Treatment group	Intercept ± SE	Slope ± SE
**(A) proximity**		
DMSO	0.40±0.061	−0.42±0.20
Atrazine low-dose	0.53±0.063	−0.76±0.30
Atrazine high-dose	0.41±0.060	−0.46±0.26
Ethynyl estradiol	0.29±0.071	0.05±0.45
**(B) number of courtship displays/ 10 min trial (log_10_ transformed)**		
DMSO	0.79±0.11	−0.02±0.27
Atrazine low-dose	0.62±0.11	0.17±0.30
Atrazine high-dose	0.76±0.10	−0.21±0.29
Ethynyl estradiol	0.13±0.14*	−0.02±0.50
**(C) number of mating attempts/ 10 min trial (square root transformed)**		
DMSO	2.37±0.24	0.63±0.17
Atrazine low-dose	2.77±0.25	0.78±0.18
Atrazine high-dose	2.56±0.23	0.72±0.14
Ethynyl estradiol	1.11±0.30*	0.47±0.45
**(D) number of attacks/ 10 min trial (square root transformed)**		
DMSO	1.77±0.22	0.65±0.15
Atrazine low-dose	1.86±0.23	0.87±0.12
Atrazine high-dose	1.07±0.22*	0.06±0.14*
Ethynyl estradiol	0.96±0.26*	−0.17±0.18*
**(E) number of aggressive displays/ 10 min trial (square root transformed)**		
DMSO	2.21±0.27	0.12±0.17
Atrazine low-dose	1.38±0.28	0.24±0.34
Atrazine high-dose	1.33±0.27*	0.04±0.26
Ethynyl estradiol	1.02±0.34*	0.15±0.33

*Intercepts and slopes of treatment groups that are significantly different (*P*<0.05) from those of the DMSO group.

The analyses of the effects of treatments within pairs showed that the solvent control males did not differ from control males with regard to any of the variables tested (proximity, *F*_1,24_ = 0.33, *P* = 0.57; courtship displays, *F*_1,24_ = 0.61, *P* = 0.44; mating attempts, *F*_1,12_ = 0.31, *P* = 0.59; attacks, *F*_1,12_ = 0.02, *P* = 0.89; aggressive displays, *F*_1,21_ = 0.00, *P* = 0.94), and neither did the atrazine low-dose males (proximity, *F*_1,22_ = 2.97, *P* = 0.10; courtship displays, *F*_1,21_ = 0.51, *P* = 0.48; mating attempts, *F*_1,11_ = 0.04, *P* = 0.85; attacks, *F*_1,11_ = 0.40, *P* = 0.54; aggressive displays, *F*_1,22_ = 0.01, *P* = 0.93). The atrazine high-dose males showed lower responses than their paired control males with respect to variables of aggression (attacks, *F*_1,23.4_ = 5.41, *P* = 0.03; aggressive displays, *F*_1,26_ = 11.15, *P* = 0.0025) but not the variables of mating effort (proximity, *F*_1,26_ = 0.34, *P* = 0.56; courtship displays, *F*_1,26_ = 0.83, *P* = 0.37; mating attempts, *F*_1,13_ = 0.04, *P* = 0.84). The ethynyl estradiol males showed lower responses than their paired control males for almost all variables measured (proximity, *F*_1,13_ = 3.50, *P* = 0.08; courtship displays, *F*_1,12_ = 47.56, *P*<0.0001; mating attempts, *F*_1,10.5_ = 4.96, *P* = 0.05; attacks, *F*_1,13_ = 13.22, *P* = 0.003; aggressive displays, *F*_1,12_ = 31.74, *P* = 0.0001).

## Discussion

### Differential susceptibility to atrazine

Population of origin did not affect mortality rates, suggesting that guppies from the different populations were not differentially impacted. Atrazine treatments did not influence mortality rates. However, estradiol can be toxic [64], [65], [66], and ethynyl estradiol may have moderately increased mortality in this study, though the trend was not statistically significant.

Although the different populations would vary naturally in the intensity and area of orange [41], it is not surprising that they did not respond differently to the treatments, because the response variable analyzed was the change in these variables over the exposure period. On the other hand, the number of courtship displays was influenced by population of origin; it is well known that guppies from different populations display at different rates [41], [67], [68]. Similarly, display rates and proximity of the focal male in relation to that of the paired control male were influenced by population of origin. This appears to be an artifact of the inherent difference in courtship intensity between high predation and low predation sites [41], [67], [68]. Possibly, in the low predation sites, individuals are more conspicuous in their competitiveness and respond to high displaying competitors by also displaying more. But in high predation sites, individuals may be more cautious in responding similarly. Interestingly, the number of mating attempts in the presence or absence of competitors was not influenced by population of origin. Perhaps because sneak copulations are less conspicuous than courtship displays [67], males in any predation regime would perform these at comparable rates; however, this may not always be the case [68]. But it must be noted that the fish in this study had been raised in the absence of predators for a few generations, and some plasticity may account for the lack of anti-predatory behaviors.

### Impaired mating signals and implications for sexual selection

As seen in other studies examining the effects of EDCs on sexual traits [26], [43], [45], [50], [53], [69], [70], [71], [72], [73], prolonged atrazine exposure reduced courtship display rates, and there was a trend for reduced expression of ornament size. The high dose of atrazine reduced the area of orange by 1%; this can alter female responses to male displays [74] such that his reproductive success is significantly reduced by two matings [75]. Area of orange is a highly heritable trait in guppies [41], and any reduction in the area must be due to reduced allocation of carotenoids to the orange spots. Though the preference for orange color varies across populations [76], female guppies generally show a preference for brighter males performing more courtship displays [41], [46], [77], and these appear to be honest signals of mate quality [78], [79], [80], [81]. In this study, the number of courtship displays was not related to the proportion of body area covered by orange; but color was associated with mating behaviors in other ways (results not shown): a composite variable including the number of courtship displays and gonopodium swings was moderately correlated with the corrected blue channel, B′, a measure of intensity of the orange spots, indicating that color intensity was associated with displays. The number of mating attempts was negatively, albeit weakly, related to the proportion of body area covered by orange, suggesting that less colorful males tended to use sneaker strategies more frequently than more colorful males.

It is particularly interesting that the behavior most affected by atrazine exposure was one believed to be an honest mating signal. Several studies (such as [82], [83], [84] among others) indicate that sex hormones play an important role in maintaining the honesty of such signals via immuno-suppressing mechanisms: increased testosterone required for the maintenance of sexual signals can damage the immune system, and individuals with an already compromised immunocompetence would be unable to signal effectively [85]. Other mating strategies like forced and sneaky copulations may be governed more by factors such as population sex ratios [86], [87], predation risk [88] and dominance hierarchies [89]; it is unclear whether sex hormones play a role in the expression of these behaviors in any species, with the exception of one example [90]. In this study, the number of mating attempts was not affected by atrazine exposure. Forced copulatory attempts in guppies are not always successful [47] and are under selection pressure via male-male competition [86] and predation [88]. These patterns then raise the question whether environmentally altered hormone levels could affect the honesty of mating signals, and whether alternate mating strategies might become more dominant in populations impacted by EDCs [9]. Experiments testing such ideas would be valuable contributions to the fields of ecotoxicology and evolutionary biology.

A few studies have analyzed the effects of EDCs on male competitive behaviors [91], [92], [93], [94]. Male-male competition is high in many species, and an individual's aggression levels can influence his access to mates [95], [96]. Pollutants are often unequally distributed across landscapes and within habitats. It is thus reasonable to expect individuals who have been impacted differently to compete against each other, especially in species that are migratory or that converge at breeding sites. The results of this study show that atrazine-impaired males in such cases may be at a mating disadvantage compared to those exposed less or not at all. Interestingly, in the presence of a rival male, the measures of mating effort (proximity to the female, number of courtship displays and number of mating attempts) were altered relatively little by atrazine exposure, but aggression was strongly reduced. I observed that when competing, the two males focused more on aggression and less on mating effort; as a result, treatment effects were stronger for the variables of aggression than for the variables of mating effort. It is pertinent to note that the difference between competing males in body area covered by orange did not influence any competitive behaviors, while differences in body size influenced only the number of mating attempts.

Aggressive displays are employed by animals to discourage the rival from attacking or competing for the resource, thereby circumventing active combat [97]. During behavioral trials, I observed that aggressive displays by one individual did not necessarily provoke aggressive displays by the other; however, attacks by one individual provoked a responding attack from the other, resulting in active fighting. Thus, I did not find a relationship between the number of aggressive displays by the focal and opponent males, but I did detect this relationship in the case of attacks, and atrazine exposure reduced the strength of the relationship. The paired control male's identity influenced the focal male's responses, suggesting that some individuals elicited stronger aggression than others. Despite this effect, the treatments had a significant effect on aggression levels. Further experiments testing whether the reduced aggression translates to reduced reproductive success would be informative. Also, it is important to know whether the EDC-altered aggression levels affect stress of exposed individuals [98], thereby influencing survival and self maintenance.

Altered mating signals and behaviors can influence population dynamics in many ways. An increased number of unattractive males in the population would alter the effective sex ratio, as females of many species, including guppies, exercise strong mate preference for sexual traits. A reduction in attractive males can also influence extra-pair mating rates [99], which can in turn alter offspring quality, disease transmission rates and predation risk [100]. EDC-altered sexual traits may not correlate with mate quality thus blurring the relationship between mate quality and signal; this can lead to females making “incorrect” mate choices that reduce their offspring quality and number [9]. These and other impacts of altered mate choice on population dynamics have been reviewed by Quader [100].

Understanding the population level effects of EDC-altered mating signals is important to conservation biology. Many contaminants are persistent and remain in the environment at substantial concentrations for several years [101], spanning multiple generations of short-lived species. Multi-generational disruption of sexual traits can alter evolutionary trajectories [9]. Future studies that aim to assess the evolutionary effects of altered sexual traits as a result of pollution must evaluate the longterm ecological consequences of chronic and persistent contamination.

### Atrazine

Several studies of sub-lethal effects of atrazine have demonstrated estrogenic effects [33], [102] and negative impacts on measures of reproduction, including fecundity, gonadal morphology, sperm counts, and hormone production [35], [36], [38], [102], [103], [104]; Rohr and McCoy [39] have reviewed several such studies. A few studies have also examined the effects of atrazine exposure on secondary sexual traits: Hayes and colleagues found that larval exposure to low doses of atrazine reduced larynx size [36] and structure [35] in African clawed frogs. The larynx is important for vocalization, the primary mating signal in many anuran species; males with smaller larynxes produce suboptimal calls. However, there is still a dearth of literature on the effects of atrazine on sexual traits. The current study advances this issue and should encourage further focus on these key effects.

The low dose of atrazine affected only courtship display rates, and not any of the other variables measured, indicating that at this concentration (a minimum of 0.26 ppb), not all mating signals are impaired in guppies. Whether this concentration may affect mating signals in other species remains to be tested; as mentioned earlier, African clawed frog larvae exposed to atrazine concentrations ranging from 1–200 ppb showed reduced larynges at metamorphosis [36]. Where there was an effect of atrazine, especially the high dose, the direction of the effect was similar to that of ethynyl estradiol suggesting that at higher doses clear estrogenic patterns may have arisen. It must be kept in mind that non-sexual behaviors were not measured in this study and so it is possible that the effects of atrazine on sexual behaviors may be due to poor health in general. Regardless, the impacts on sexual traits seen here are significant enough to be of concern. Dose-response studies with a larger range of atrazine concentrations would help determine the concentrations and exposures influencing different end-points in wildlife species. Understanding the effects on sexual traits is especially important because of their subtle yet crucial implications for reproduction and populations dynamics. More studies along these lines will highlight the negative impacts of atrazine on wildlife reproduction. There may be similar effects on human health as well, because the mechanism of action of atrazine is similar across most vertebrate taxa, including humans [102].
